# Sensor-Based Hand and Upper-Limb Movement Intention Recognition in Post-Stroke Rehabilitation: A Scoping Review

**DOI:** 10.3390/s26216680

**Published:** 2026-10-22

**Authors:** Jiwon Shin, Wonjun Oh, Youngjoo Cha

**Affiliations:** Department of Physical Therapy, Cheju Halla University, 38 Halladaehak-ro, Jeju 63092, Republic of Korea; shinjw12@chu.ac.kr (J.S.); june01047@chu.ac.kr (W.O.)

**Keywords:** stroke, upper extremity, movement intention, brain–computer interfaces, electromyography, wearable sensors, rehabilitation, scoping review

## Abstract

Background: Sensor-based systems increasingly allow post-stroke rehabilitation devices to infer voluntary movement intention and respond to active effort, but approaches vary widely in sensor modality, target movement, recognition method, and application. This review mapped sensor-based hand and upper-limb intention recognition after stroke. Methods: Following the Population–Concept–Context framework and PRISMA-ScR, PubMed, Embase, CINAHL, MEDLINE, and PEDro were searched from inception to 21 July 2026, and IEEE Xplore was additionally searched on 24 September 2026. Studies using sensor-based systems to classify, decode, trigger, assess, or provide feedback on voluntary hand or upper-limb movement in adults with stroke were included and narratively synthesized. Results: Of 1576 records, 17 studies were eligible: 5 electromyography (EMG)-based or myoelectric systems, 7 electroencephalography (EEG)/brain–computer interface (BCI)-based systems, 2 EMG-triggered functional electrical stimulation (FES) systems, and 3 multimodal assessment or feedback systems. Target movements were mainly hand opening/closing, grasping, and finger, wrist, or reaching movements. Applications ranged from intention-related physiological assessment and movement/performance feedback to real-time closed-loop control of robots, orthoses, exoskeletons, and FES. True multimodal sensor fusion was uncommon. Conclusions: Sensor-based intention-responsive rehabilitation after stroke has developed mainly through parallel EMG-based and EEG/BCI-based approaches, with limited multimodal integration and no standardized framework. The present review distinguishes direct intention decoding, sensor-triggered control, movement/performance feedback, and intention-related physiological assessment; future work should apply these distinctions consistently and advance multimodal integration and clinical validation.

## 1. Introduction

Stroke remains a major global cause of death and disability. Recent global estimates indicate that stroke is the third leading cause of death and disability combined worldwide, with approximately 11.9 million new stroke cases in 2021 and a continuing increase in stroke-related disability burden [1]. Among the various consequences of stroke, upper-limb motor impairment is particularly important because it directly affects independence, self-care, and participation in daily life. Up to 85% of stroke survivors experience some degree of upper-limb paresis, and approximately half continue to have impaired upper-limb or hand function in the chronic phase [2]. Persistent arm and hand dysfunction commonly limits activities such as reaching, grasping, eating, dressing, and washing, making upper-limb recovery a central target of post-stroke rehabilitation.

Conventional upper-limb rehabilitation after stroke commonly relies on repetitive task practice, therapist-guided exercise, neuromuscular re-education, and functional training [3]. Although these approaches remain essential, they can be limited by therapist availability, variability in movement quality, insufficient intensity, and difficulty providing precise real-time feedback. In response, technology-assisted rehabilitation has increasingly been explored to support repetitive, intensive, and feedback-driven training. Robotic devices, functional electrical stimulation (FES), orthoses, exoskeletons, virtual environments, wearable sensors, and brain–computer interface (BCI) systems have all been investigated as ways to augment upper-limb recovery after stroke. Recent reviews have highlighted the expanding use of technology-enhanced rehabilitation, including BCI-controlled electrical stimulation [4] and wearable sensor-based approaches for upper-extremity assessment and treatment after stroke [5].

Real-time technology guidance in post-stroke upper-limb rehabilitation also includes ultrasound imaging, which can improve anatomical localization and intervention precision during spasticity management [6,7]. This imaging-guided application is clinically relevant but conceptually distinct from movement-intention recognition because it supports anatomical assessment and treatment targeting rather than decoding voluntary motor intent.

A key component of technology-assisted rehabilitation is the ability of a system to detect or infer the user’s voluntary movement intention. Sensor-based movement intention recognition may allow rehabilitation devices to respond to the user’s active effort rather than delivering passive or pre-programmed assistance. For example, electromyography (EMG) can be used to detect residual muscle activation [8], electroencephalography (EEG)-based BCIs can identify motor imagery or movement-related brain activity [9], and motion or force sensors can provide information about movement execution, compensation, or task performance. These signals can be used to trigger electrical stimulation, drive robotic assistance, control hand orthoses, provide feedback, or classify intended hand and upper-limb movements. Such closed-loop or intention-responsive systems may be particularly relevant for promoting active participation and sensorimotor integration during post-stroke rehabilitation.

For conceptual clarity, this review distinguishes four functional roles of sensing: (1) intention decoding, in which sensor signals are used to infer an intended movement or motor state; (2) sensor-triggered control, in which detected voluntary activity initiates or modulates a rehabilitation device or intervention; (3) movement/performance feedback, in which sensor-derived information regarding executed movement, compensation, or task performance guides rehabilitation; and (4) intention-related physiological assessment, in which physiological signals associated with voluntary upper-limb tasks characterize task-related responses without directly controlling a rehabilitation device.

Despite this growing interest, the literature on sensor-based intention recognition in post-stroke upper-limb rehabilitation remains heterogeneous. Studies differ widely in the sensor modalities used, target movements, algorithms, control strategies, rehabilitation devices, feedback approaches, participant populations, and validation methods. Some studies focus on EMG-driven or myoelectric control, whereas others use EEG-based BCIs, FES, robotic hand devices, wearable interfaces, or multimodal feedback systems. However, many technology-based rehabilitation studies report clinical effects without clearly describing intention-recognition mechanisms, while many engineering studies focus on signal classification or device control without sufficient clinical rehabilitation context. As a result, it remains unclear how sensor-based intention recognition has been implemented across post-stroke upper-limb rehabilitation studies and how far the field has progressed toward multimodal sensor integration.

Previous reviews have examined related areas, such as wearable sensors for upper-extremity assessment and treatment after stroke [7], BCI-controlled electrical stimulation for upper-limb rehabilitation [6], and robot-assisted upper-limb rehabilitation [10]. However, these reviews have generally focused on specific technologies, therapeutic effects, or assessment methods. To our knowledge, the broader landscape of sensor-based hand and upper-limb movement intention recognition in post-stroke rehabilitation has not been sufficiently mapped. In particular, there is a need to clarify which sensor modalities are used, what types of hand or upper-limb movements are targeted, which recognition or control approaches are applied, whether systems are tested offline or in real time, and whether they are integrated with rehabilitation robots, orthoses, electrical stimulation, or feedback-based training.

Therefore, this scoping review aims to systematically explore and map sensor-based approaches used for hand or upper-limb movement intention recognition in post-stroke rehabilitation. Specifically, this review identifies the sensor modalities, target movements, algorithms, participant characteristics, validation methods, real-time applicability, integration with rehabilitation robots or hand orthoses, level of rehabilitation application, and extent of multimodal sensor integration. By mapping these characteristics, this review seeks to clarify current research trends and identify evidence gaps that may guide future development of sensor-driven, intention-responsive rehabilitation systems for stroke survivors.

## 2. Methods

### 2.1. Study Design

This scoping review was conducted to map the existing literature on sensor-based hand and upper-limb movement intention recognition in post-stroke rehabilitation. A scoping review design was selected because the purpose of this review was not to determine intervention effectiveness, but to identify the range of sensor-based approaches, system characteristics, rehabilitation applications, and evidence gaps in an emerging and heterogeneous research area.

The review was guided by the methodological framework for scoping reviews and reported in accordance with the Preferred Reporting Items for Systematic Reviews and Meta-Analyses extension for Scoping Reviews (PRISMA-ScR) [11]. The protocol for this scoping review was retrospectively registered in the Open Science Framework (OSF; https://osf.io/4aku9 (accessed on 28 July 2026)).

### 2.2. Eligibility Criteria

Eligibility criteria were developed according to the Population–Concept–Context framework [12]. The detailed inclusion and exclusion criteria are presented in Table 1.

### 2.3. Information Sources and Search Strategy

Studies eligible for this review were identified through electronic searches of PubMed, Embase, CINAHL, MEDLINE, and PEDro from database inception to 21 July 2026. Grey literature was excluded from the search process. The search strategy combined terms related to stroke, upper-limb or hand function, and sensor-based intention recognition or control. Search terms included “stroke,” “post-stroke,” “cerebrovascular accident,” “hemiplegia,” or “hemiparesis”; “upper limb,” “upper extremity,” “arm,” “hand,” “wrist,” “finger,” “grasp,” or “reach”; and “EMG,” “sEMG,” “myoelectric,” “EEG,” “BCI,” “sensor,” “wearable sensor,” “IMU,” “camera,” “Kinect,” “Leap Motion,” “robot,” “orthosis,” “exoskeleton,” “functional electrical stimulation,” “intention recognition,” “motor intention,” “movement classification,” “gesture recognition,” “movement decoding,” “volitional control,” “sensor fusion,” or “data fusion.” Search strategies were adapted for each database according to database-specific syntax and indexing terms. In response to peer review, IEEE Xplore was additionally searched on 24 September 2026 using stroke, upper-limb, sensor/robotics/intention-recognition, and randomized-trial concepts to strengthen coverage of engineering, robotics, BCI, signal-processing, and sensor-fusion literature. The IEEE Xplore search yielded 33 records. Search results were exported to Microsoft Excel, and duplicate records were removed before title and abstract screening. No additional identification methods, including manual reference-list screening, were used. The original biomedical and rehabilitation database searches were completed on 21 July 2026, whereas the supplementary IEEE Xplore search was completed on 24 September 2026. Thus, these dates refer to separate search stages rather than a single common search cutoff. Supplementary Table S1 provides the full database-specific strategies, including field-tag and indexing adaptations for the biomedical databases, the broader manually screened PEDro search, and the complete IEEE Xplore Boolean query with trial-related terms.

### 2.4. Study Selection

Titles and abstracts of the remaining records were independently screened by two reviewers (J.S. and W.O.) against the eligibility criteria. Reports judged potentially relevant were retrieved for full-text assessment. The selection process followed two stages. First, titles and abstracts were screened to exclude clearly irrelevant records, including studies unrelated to stroke, upper-limb rehabilitation, sensor-based systems, intention recognition, control, or feedback. Second, full texts or sufficiently detailed reports were assessed for eligibility according to the Population–Concept–Context criteria. Reports were excluded at the full-text stage if they did not report outcomes related to movement intention recognition, movement classification, motor decoding, device control, sensor-based feedback, or rehabilitation application; if the intervention or system was not relevant to sensor-based upper-limb intention recognition or control; or if the study design was ineligible.

All 49 full-text reports were independently assessed by each of the two reviewers. Agreement was reached for 46 of the 49 full-text reports (93.9%; Cohen’s κ = 0.830). Disagreements regarding the remaining three reports were resolved through discussion and consultation with a third reviewer (Y.C.) based on the predefined eligibility criteria. Following resolution, 17 studies met all eligibility criteria and were included in the final review.

### 2.5. Data Charting

Data were charted by one reviewer (J.S.) using a standardized extraction form and independently verified by a second reviewer (W.O.). Classifications requiring interpretive judgment, including rehabilitation application level, multimodal sensor integration level, and the functional role of sensing, were independently reviewed by J.S. and W.O. Discrepancies were resolved through discussion and, when necessary, consultation with a third reviewer (Y.C.). The following information was extracted:

- author and year of publication;
- country;
- study design;
- participant characteristics;
- stroke stage, if reported;
- sample size;
- sensor modality;
- device or rehabilitation system;
- control or recognition approach;
- target movement or task;
- algorithm or signal-processing method;
- validation method;
- performance or clinical outcome;
- real-time applicability;
- robot, orthosis, FES, or feedback integration;
- rehabilitation application level;
- multimodal sensor integration level;
- main findings;
- key limitations reported by the authors.

Sensor modalities were categorized as EMG-based, EEG/BCI-based, EMG-triggered FES or game-based systems, multimodal assessment or feedback systems, and robot/orthosis-integrated systems.

### 2.6. Data Synthesis

A descriptive synthesis was conducted. Included studies were summarized in tables and grouped according to sensor modality, control or recognition approach, target movement, and rehabilitation application level.

For synthesis, sensor use was additionally classified as intention decoding, sensor-triggered control, movement/performance feedback, or intention-related physiological assessment. Sensors used only for physiological or mechanistic assessment were not interpreted as contributing to multimodal intention decoding unless their information directly contributed to recognition or device control.

The rehabilitation application level was categorized as follows:

1. offline classification or signal analysis only;
2. real-time recognition or control;
3. robot, orthosis, or FES control;
4. rehabilitation training feedback;
5. clinical rehabilitation application.

The level of multimodal sensor integration was categorized as follows:

1. single-sensor approach;
2. multiple sensors used for assessment only;
3. multiple sensors used in the same rehabilitation system;
4. true sensor fusion for intention recognition or control.

Findings were synthesized narratively to identify current research trends, commonly used sensor modalities, types of target movements, control and feedback strategies, levels of clinical application, and remaining evidence gaps. Critical appraisal of individual sources of evidence was not conducted because the purpose of this scoping review was to map the range and characteristics of the literature rather than to evaluate intervention effectiveness.

## 3. Results

### 3.1. Study Selection

The database searches identified 1576 records, including 254 from PubMed, 463 from Embase, 189 from CINAHL, 174 from MEDLINE, 463 from PEDro, and 33 from IEEE Xplore. After removal of 265 duplicate records, 1,311 records were screened by title and abstract. Of these, 1,237 records were excluded because they were not related to post-stroke upper-limb rehabilitation, sensor-based systems, movement intention recognition, device control, or rehabilitation feedback.

**Figure 1:**
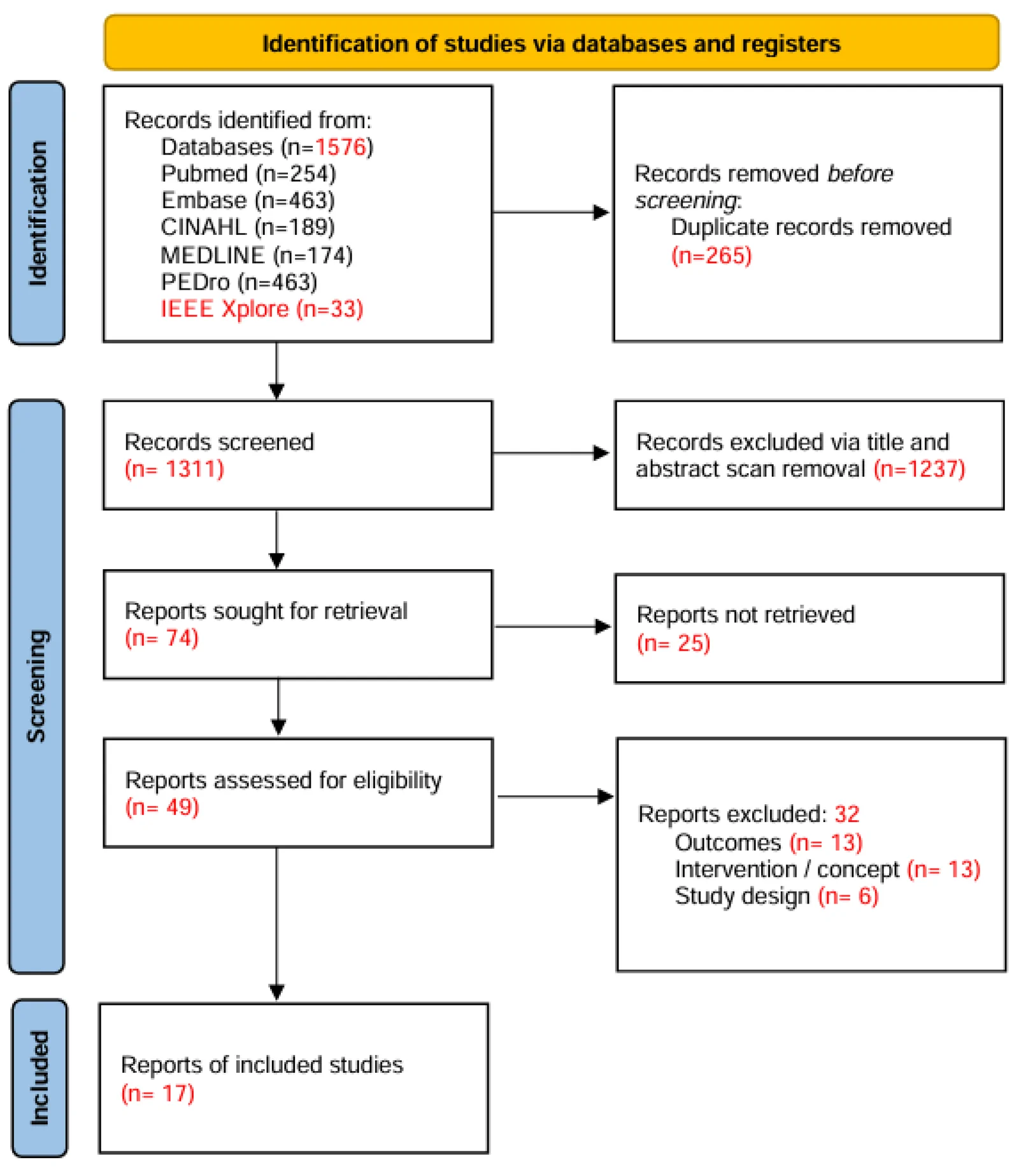
PRISMA flow diagram of the study selection process. Adapted from the PRISMA 2020 flow diagram template [13], which is distributed under the Creative Commons Attribution (CC BY 4.0) license.

A total of 74 reports were sought for retrieval. Twenty-five reports could not be retrieved, and 49 reports were assessed for eligibility. At the full-text eligibility stage, 32 reports were excluded: 13 because the outcomes were not relevant to movement intention recognition, control, or sensor-based feedback; 13 because the intervention or concept was not relevant to sensor-based upper-limb intention recognition, assistive control, or task-related feedback; and 6 because of ineligible study design. Finally, 17 studies were included in this scoping review. The study selection process is presented in Figure 1.

### 3.2. General Characteristics of Included Studies

Seventeen studies were included in this scoping review [14,15,16,17,18,19,20,21,22,23,24,25,26,27,28,29,30]. The included studies investigated a range of sensor-based approaches for detecting, decoding, triggering, assessing, or providing feedback on voluntary hand and upper-limb movement in post-stroke rehabilitation contexts.

The studies were grouped according to the primary sensor modality or system type. Five studies were categorized as EMG-based or myoelectric systems [14,16,20,22,24]. Seven studies were categorized as EEG/BCI-based systems [17,19,21,23,25,27,28]. Two studies used EMG-triggered FES systems [18,29], and three studies were categorized as multimodal assessment or feedback systems [15,26,30]. An overview of the included study categories is provided in Table 2.

The main characteristics of the included studies are summarized in Table 3. Study IDs (S1–S17) were assigned for presentation purposes and do not correspond to the order of database searching.

### 3.3. Sensor Modalities and System Types

EMG-based and myoelectric systems were the most common sensor-based approaches identified in this review. These studies used residual muscle activity or EMG patterns to trigger stimulation, control assistive devices, support robotic or orthotic movement, or provide interactive training feedback. These systems were generally designed to promote active user participation by linking voluntary muscle activation to device movement, feedback, or task performance.

EEG/BCI-based systems formed another major group of included studies. These studies used brain signals, most commonly motor imagery or movement-related EEG activity, to control robotic devices, hand orthoses, exoskeletons, or stimulation-assisted rehabilitation systems. Several EEG/BCI-based systems were designed to create an intention-responsive rehabilitation environment, in which detected motor intention could trigger or guide hand and upper-limb training.

A small number of studies incorporated more than one sensor or feedback modality. These included systems combining physiological signals, motion tracking, force information, functional electrical stimulation, pressure sensing, RFID-tagged objects, or visual and social feedback. However, most included studies relied on a single primary sensor modality, particularly EMG or EEG/BCI. True multimodal sensor fusion for movement intention recognition or assistive control was limited.

The three studies newly identified through IEEE Xplore further illustrated this range. Guo et al. used SSVEP classification to generate robotic-glove commands [28]; Ambrosini et al. combined threshold-based EMG-triggered FES with exoskeleton angle sensing and RFID-based task monitoring [29]; and Feingold-Polak et al. used sensorized objects and kinematic measures to support socially assistive, task-oriented feedback without direct intention decoding [30].

Accordingly, S2 was interpreted as intention-related physiological assessment rather than direct intention decoding, whereas S13 was interpreted as movement/performance feedback rather than a movement-intention decoding system.

The sensor, system, control, and task characteristics of the included studies are summarized in Table 4.

### 3.4. Control, Feedback, and Rehabilitation Application

The included studies varied in their level of rehabilitation application. Some studies focused mainly on signal classification or movement-intention detection, whereas others implemented real-time control of rehabilitation devices or sensor-derived task feedback. Several studies integrated sensor-based recognition or triggering with assistive technologies such as robotic hands, hand orthoses, exoskeletons, functional electrical stimulation, socially assistive robots, or game-based rehabilitation systems.

The target movements or tasks also varied across studies. Common targets included hand opening and closing, grasping, finger movement, wrist or hand movement, reaching-related upper-limb movement, and functional upper-limb tasks. In some studies, the sensor-based system was used to detect intended movement directly, whereas in others the system provided feedback on movement quality, compensatory movement, or task performance.

Closed-loop implementation varied across studies. Threshold-based detection, trained classification, and continuous signal-to-feedback mapping were used to trigger or guide robotic, orthotic, FES, or game-based systems. However, technical performance measures such as recognition accuracy, response latency, and online adaptability were inconsistently reported, limiting direct comparison. The available technical characteristics are summarized in Table 5.

Across the included systems, the temporal and feedback characteristics were not directly comparable. For example, S7 used a 50-ms EMG averaging window for continuous cursor feedback, whereas S15 used 3-s EEG analysis windows with 0.25-s updates and a four-consecutive-detection rule to issue robotic-glove commands. These values describe signal-processing or decision parameters and should not be interpreted as measured end-to-end response latency. Threshold-triggered FES systems (e.g., S5 and S16) linked detected muscle activity to stimulation, whereas EEG/BCI systems (e.g., S4, S10, and S12) relied on calibrated detection or classification to provide contingent robotic or orthotic assistance. Feedback modalities ranged from visual cursor or virtual-hand feedback to proprioceptive movement and electrical stimulation. Although S16 reported the proportion of FES tasks successfully triggered, recognition accuracy, total command-to-actuation delay, and online adaptation were not consistently quantified across studies. Consequently, the available reports do not support a quantitative ranking of closed-loop responsiveness or adaptability (Table 5).

### 3.5. Multimodal Sensor Integration

The level of multimodal sensor integration varied across the included studies. Most studies used a single-sensor approach, such as EMG-based control or EEG/BCI-based control. Some studies used multiple sensors within the same rehabilitation system, but the sensors were often used for assessment, feedback, or supplementary monitoring rather than for integrated intention recognition. Only a limited number of studies incorporated multimodal information in a way that approached sensor fusion for movement intention recognition or rehabilitation control.

Overall, the evidence suggests that sensor-based intention recognition in post-stroke upper-limb rehabilitation has been explored mainly through EMG-based and EEG/BCI-based approaches. Although multimodal systems are emerging, the integration of multiple sensor modalities for real-time intention recognition, adaptive control, and clinically meaningful rehabilitation feedback remains limited.

## 4. Discussion

The purpose of this scoping review was to map how sensor-based approaches have been used to detect, decode, or respond to voluntary hand and upper-limb movement intention in post-stroke rehabilitation and to identify current trends and evidence gaps in this developing field. Across the 17 included studies, sensor-based intention-responsive rehabilitation was implemented mainly through two distinct paradigms, EMG-based or myoelectric systems and EEG/BCI-based systems, together with a smaller number of systems incorporating EMG-triggered FES and multimodal assessment or feedback. The three additional IEEE Xplore studies broadened the mapped evidence to include SSVEP-based robotic-glove control, a hybrid EMG-triggered FES/exoskeleton platform with sensorized task monitoring, and socially assistive robot-guided upper-limb training. Rather than converging on a single dominant technology, these findings point to a field that is technically active but still fragmented, with sensor choice, target task, control strategy, and level of clinical integration varying considerably across studies.

The refined framework distinguishes direct intention decoding from sensor-triggered control, movement/performance feedback, and intention-related physiological assessment. This distinction allows S2 and S13 to remain informative to the broader rehabilitation context without being interpreted as direct intention-decoding systems.

The distribution of EMG-based and EEG/BCI-based systems appears to reflect two different rehabilitation logics rather than a clear consensus on how movement intention should be sensed. EMG-based systems relied on residual peripheral muscle activity, obtained from either the paretic limb or, in some systems, the non-paretic side, and were used to drive myoelectric orthoses, trigger FES, or provide real-time biofeedback. Their reliance on detectable muscle activity suggests that the feasibility of these approaches may depend partly on the presence and reliability of residual or contralateral volitional muscle signals. EEG/BCI-based systems, in contrast, decoded central sensorimotor activity associated with motor imagery or attempted movement and may therefore provide an alternative pathway when peripheral muscle signals are limited or unreliable. However, most EEG/BCI systems required prior calibration, signal processing, or classifier training before online use, and the extent of genuine real-time closed-loop control varied across studies. Taken together, these patterns suggest that sensor modality selection may be influenced by the availability and reliability of peripheral or central motor signals rather than reflecting convergence on a single dominant recognition paradigm. Although clinically plausible, this interpretation requires further investigation because the included studies were not designed to directly compare sensor-selection strategies across levels of motor impairment.

Target movements were relatively narrow, clustering around hand opening and closing, grasping, and finger or wrist movements, with fewer studies addressing more proximal or functional multi-joint tasks. This distal emphasis may reflect the clinical importance of restoring hand function in individuals with substantial upper-limb impairment; however, it also means that the reviewed systems address only part of the functional upper-limb movement repertoire required for daily activities such as reaching, arm transport, and object manipulation. Rehabilitation applications ranged from sensor-based movement detection and real-time feedback to closed-loop control of robots, orthoses, exoskeletons, or FES within structured training paradigms. Several studies also compared intention-driven systems with sham, passive, or non-contingent control conditions, providing a framework for examining the contribution of intention-contingent feedback separately from repetitive practice or device-assisted movement alone. Nonetheless, only a minority of studies examined sustained clinical use or longer-term outcomes, suggesting that many systems remain at proof-of-concept, feasibility, or early clinical-evaluation stages rather than established clinical tools.

A related engineering study by Liu et al. [31] developed a Newton–Euler model of a human–machine coupled shoulder–elbow system that incorporated elastic-strap constraints, exoskeleton friction, and time-varying joint impedance. The authors used an optimized short-time linear time-varying parameter-identification method with QR decomposition and a wearable exoskeleton with inertial sensors to estimate joint stiffness and damping. In experiments involving three adult male wearers, joint-torque reconstruction errors ranged from 1.67 to 2.84 N·m (RMSE). Although the platform collected kinematic and dynamic measurements in real time, these results describe impedance estimation and torque reconstruction, not movement-intention decoding, online intention-classification accuracy, or end-to-end closed-loop response latency. Moreover, the experiments were not conducted in a post-stroke population. Accordingly, this article informs the engineering context of human–robot interaction but does not meet the Population–Concept–Context eligibility criteria and is not counted among the 17 included rehabilitation studies.

A central finding of this review is that true multimodal sensor fusion for movement intention recognition remains limited. Although several studies incorporated more than one sensing or feedback modality, including combinations involving EEG, EMG, functional near-infrared spectroscopy (fNIRS), motion tracking, force sensing, or other physiological and kinematic measures, the additional modalities were often used for assessment, monitoring, mechanistic evaluation, or feedback rather than contributing directly to the intention-recognition algorithm or device-control loop. Thus, the presence of multiple sensors within a rehabilitation system should not necessarily be interpreted as true sensor fusion. Only a limited number of systems integrate information from multiple sources in a manner approaching combined real-time control or adaptive feedback. This gap is notable because multimodal fusion may potentially compensate for limitations of individual signal sources and improve robustness when EMG or EEG signals are weak, variable, or susceptible to artifact. At the same time, integrating multiple modalities introduces challenges related to synchronization, signal processing, computational complexity, calibration, and clinical usability [32]. These technical and practical demands may partly explain the continued predominance of single-modality systems.

The included studies illustrate different degrees of algorithmic complexity: S1 employed an AI-integrated, calibrated EMG-pattern recognition approach, S12 applied common spatial pattern features and linear discriminant analysis for EEG-based motor-attempt classification, and S15 used canonical correlation analysis for steady-state visually evoked potential recognition. In contrast, S3, S5, and S16 primarily used signal features or individualized thresholds to trigger or modulate assistance. These examples demonstrate methodological diversity, but do not establish that learning-based methods outperform threshold-based approaches, because populations, tasks, feedback configurations, and technical reporting differed. Future studies should evaluate robustness to inter-session signal drift and explicitly report recalibration and adaptation procedures.

Artificial intelligence and machine learning methods may improve the robustness and adaptability of movement-intention recognition, particularly when EMG or EEG characteristics vary across participants, sessions, or rehabilitation stages. Future evaluations should report model training and validation, cross-session stability, participant-specific adaptation, response latency, and generalization to clinically meaningful upper-limb tasks.

The participant and study-design characteristics of the included studies further contextualize the current state of the field. Study populations represented different stages of stroke recovery, including post-acute, subacute, and chronic populations, and sample sizes were generally modest. Several studies used randomized, sham-controlled, or crossover designs intended to distinguish intention-contingent control from passive or non-contingent device assistance, whereas one newly included study provided an early uncontrolled clinical evaluation of a hybrid robotic system. This pattern suggests that the field is beginning to move beyond primarily engineering-focused validation toward more structured clinical evaluation. However, heterogeneous participant characteristics, modest sample sizes, and generally short intervention periods limit the generalizability of these findings and preclude conclusions regarding longer-term functional recovery. Moreover, because this scoping review was designed to map technological and rehabilitation characteristics rather than evaluate treatment effectiveness, no conclusions can be drawn regarding the comparative clinical efficacy of individual sensor modalities or control approaches.

These findings extend previous reviews that have examined wearable sensing [5], BCI-controlled electrical stimulation [4], or robot-assisted upper-limb rehabilitation [10] as relatively distinct technological domains. By considering sensor modality, target movement, recognition or control strategy, rehabilitation application, and multimodal integration within a common framework, the present review provides a broader view of how movement-intention recognition is embedded within post-stroke upper-limb rehabilitation systems rather than treating sensing and rehabilitation devices as separate technological areas. Despite the diversity of devices and platforms represented in the literature, the underlying sensing and control logic appears to converge on a relatively small number of recurring patterns, including detection of residual muscle activity, decoding of central motor-related activity, intention-contingent device assistance, and sensor-based feedback. At the same time, substantial variation in how movement intention recognition is defined and operationalized indicates that technological development has not yet been accompanied by a standardized conceptual or methodological framework.

Several limitations of this review should be considered. First, consistent with scoping review methodology, no formal methodological quality or risk-of-bias assessment of the included studies was conducted. Accordingly, this review describes the range and characteristics of the literature rather than the strength or certainty of evidence regarding clinical effectiveness. Second, although the search covered five biomedical/rehabilitation databases and was supplemented by IEEE Xplore, grey literature was excluded, which may have omitted relevant conference proceedings, preprints, engineering reports, or other non-indexed studies, particularly early-stage engineering work on multimodal sensor integration and advanced classification methods that may first appear in conference or non-journal formats. Third, the relatively small number of included studies, several of which were feasibility or pilot investigations, limits the generalizability of the observed patterns. In addition, definitions and implementations of movement intention recognition varied substantially across studies. The boundary between direct intention decoding, sensor-triggered device control, physiological assessment, and sensor-based rehabilitation feedback was not always clearly defined, which may have influenced study classification and complicated comparison across systems. Fourth, the review protocol was registered retrospectively in the Open Science Framework rather than prospectively before the review process began. As a result, the registration did not provide a prospective, time-stamped record of the planned methods, and the possibility of post-hoc methodological decisions cannot be fully excluded. This should be considered when interpreting the methodological transparency and reproducibility of the review.

Building on these findings, future research may benefit from greater standardization in reporting sensor characteristics, preprocessing methods, classification or control algorithms, validation procedures, real-time performance, and clinical outcomes. More consistent reporting would facilitate comparison across technologies and help clarify which approaches may be appropriate for different levels of post-stroke motor impairment. Further investigation of true multimodal sensor fusion is also warranted, particularly approaches that integrate complementary peripheral and central signals or combine physiological signals with kinematic and force information within a common intention-recognition framework. Expanding target movements beyond isolated hand opening, grasping, and finger or wrist movements toward more functional multi-joint upper-limb tasks may also improve ecological and clinical relevance. Finally, larger prospective studies with longer intervention periods and clinically meaningful follow-up are needed to determine whether intention-responsive closed-loop systems provide additional rehabilitation value beyond conventional repetitive training or non-contingent device assistance. Addressing these methodological and translational gaps may help move the field from technically feasible intention-recognition systems toward more robust, generalizable, and clinically usable rehabilitation technologies.

## 5. Conclusions

Sensor-based movement intention recognition in post-stroke upper-limb rehabilitation has developed primarily through EMG-based and EEG/BCI-based approaches, while true multimodal sensor fusion remains limited. Target movements have largely focused on hand and distal upper-limb tasks, and this review distinguishes intention decoding, sensor-triggered control, movement/performance feedback, and intention-related physiological assessment to clarify these previously inconsistent conceptual boundaries. Future research should establish clearer definitions, advance multimodal integration, and evaluate intention-responsive systems using more functional movements and clinically meaningful outcomes.

## Figures and Tables

**Table 1:** Inclusion and exclusion criteria based on the Population–Concept–Context framework.

Element	Inclusion Criteria	Exclusion Criteria
Population(P)	Adults aged ≥ 18 years. Medically diagnosed with stroke (Ischemic or Hemorrhagic). Post-stroke upper-limb motor impairment (Acute, Subacute, or Chronic stages). Healthy adults included if the system is explicitly for stroke rehabilitation.	Children and adolescents (<18 years). Other neurological conditions (TBI, SCI, etc.) as a primary focus. Mixed clinical populations where stroke-specific data cannot be extracted.
Concept(C)	Sensor-based intention recognition: Systems using sensors (sEMG, EEG, IMU, Camera, Force) to classify or decode voluntary movement intention. Real-time control & feedback: Myoelectric/BCI control of robots, orthoses, exoskeletons, or FES systems. Outcomes: Studies reporting accuracy, classification performance, response time, or clinical recovery metrics (FMA, ARAT). Intention-related physiological assessment was eligible only when the measured signals were explicitly linked to a voluntary upper-limb rehabilitation task.	Studies using sensors only for pre/post-assessment or passive monitoring (no intention logic). Focus on metrics unrelated to intention (e.g., fatigue prediction only, biomechanical analysis only). Conventional manual rehabilitation without sensor integration. Passive monitoring or assessment without an explicit relationship to voluntary movement or rehabilitation-task performance remained excluded.
Context(C)	Upper limb rehabilitation settings: Hospital, rehabilitation center, laboratory-based validation for rehab, or community/home-based environments. Assistive technologies: Robotic therapy, hand orthoses, exoskeletons, wearables, and digital/VR rehabilitation.	Environments not related to stroke or upper-limb/hand rehabilitation. Assistive devices not intended for functional motor recovery.

Abbreviations: ARAT, Action Research Arm Test; BCI, brain–computer interface; EEG, electroencephalography; FES, functional electrical stimulation; FMA, Fugl-Meyer Assessment; IMU, inertial measurement unit; SCI, spinal cord injury; sEMG, surface electromyography; TBI, traumatic brain injury; VR, virtual reality.

**Table 2:** Overview of included study categories.

**Category**	**No. of Studies**
EMG-based or myoelectric systems	5
EEG/BCI-based systems	7
EMG-triggered FES systems	2
Multimodal assessment or feedback systems	3
Total	17

**Table 3:** Characteristics of included studies.

Study ID	Author, Year	Country	Study Design	Participants	Stroke Stage/ Time Since Stroke	Setting
S1	Murakami et al., 2023 [14]	Japan	Single-blind, randomized, parallel-controlled trial	20 patients with chronic stroke and moderate-to-severe upper-extremity hemiparesis (active *n* = 11; passive-robot control *n* = 9)	Chronic stroke; inclusion required >60 days after stroke onset	Outpatient clinic, Department of Rehabilitation Medicine, Juntendo University Hospital
S2	Cheng et al., 2024 [15]	China	Single-blind randomized controlled clinical trial with randomized sequence of three robot-assisted rehabilitation modes; 3-day intervention with a 1-day washout	10 patients with post-stroke hand dysfunction	2 weeks to 3 months after stroke onset	Rehabilitation Center, Shanghai Seventh People’s Hospital
S3	Zhou et al., 2017 [16]	China	Randomized controlled trial	42 patients with hemiplegia after stroke were randomized; 36 completed all assessments (EMGB *n* = 18; cyclic NMES *n* = 18)	Subacute stroke; first stroke 2 weeks to 6 months before the study	Hospital rehabilitation setting, Department of Rehabilitation Medicine, ZhongDa Hospital, Nanjing
S4	Ji et al., 2025 [17>]	China	Prospective, single-centre, two-arm parallel randomized controlled trial; described by the authors as a pilot study	40 patients with subacute stroke and left-sided hemiparesis were randomized (BCI-SRG *n* = 20; SRG *n* = 20); 39 completed clinical assessments	Subacute stroke; stroke onset within 2 weeks to 3 months	Rehabilitation Medicine Department, Wuxi Central Rehabilitation Hospital
S5	Höhler et al., 2023 [18]	Germany	Randomized crossover feasibility study	18 patients after stroke with unilateral upper-limb paresis (MRC ≤ 4) were analyzed	Subacute stroke; the investigated cohort was ≤7 months after stroke	Inpatient rehabilitation hospital, Schoen Clinic Bad Aibling
S6	Hu et al., 2021 [19]	China	Single-centre, single-blinded, prospectively randomized controlled preliminary study	12 patients with severe upper-limb and hand motor impairment after stroke (MI-BCI *n* = 7; classic MI *n* = 5)	Chronic stroke; inclusion required disease duration >2 months	Huashan Hospital, Fudan University; motor-imagery training was conducted in a fixed quiet room
S7	Mugler et al., 2019 [20]	USA	Randomized three-group trial	32 chronic stroke survivors with moderate-to-severe arm impairment; randomized to 3 training groups	Chronic stroke; ≥6 months after stroke onset	Research setting at Northwestern University/Shirley Ryan AbilityLab; exact intervention site category was not explicitly labelled in the article
S8	Ramos-Murguialday et al., 2019 [21]	Germany	Six-month long-term follow-up of a previous double-blinded controlled randomized BMI study	30 chronic stroke patients with severe hand paresis from the previous study were invited; 28 underwent follow-up (experimental *n* = 16; sham *n* = 12)	Chronic stroke; parent-study inclusion required ≥ 10 months after stroke onset and no active finger extension	BMI training setting not explicitly stated in this follow-up report; a home-based training program was provided after the intervention
S9	Willigenburg et al., 2017 [22]	USA	Randomized subgroup study conducted within a larger randomized controlled trial	12 chronic post-stroke participants with moderate upper-extremity impairment (RTP+Myomo *n* = 7; RTP only *n* = 5)	Chronic stroke; single stroke >12 months before enrollment	Outpatient/research rehabilitation context; exact intervention site was not explicitly reported
S10	Frolov et al., 2017 [23]	Russia	Randomized, blind, controlled multicenter trial	74 inpatients after ischemic or hemorrhagic stroke with hand paresis (BCI *n* = 55; control *n* = 19)	Subacute (1–6 months) or chronic (>6 months) stroke	Inpatient treatment at three medical centers with neurorehabilitation or motor-rehabilitation services
S11	Khorasani et al., 2026 [24]	USA	Single-blind, randomized, sham-controlled trial	94 participants were randomized to 3D, 2D, 2D Reach, or sham groups; 59 completed the 6-week training and were included in the final analysis	Chronic stroke; ≥6 months before enrollment; completers had mean 6.4 years since stroke	Home-based training 5 days/week plus laboratory training 1 day/week for 6 weeks; study conducted at Northwestern University
S12	Fu et al., 2023 [25]	China	Randomized controlled trial with blinded outcome assessment	66 patients after ischemic or hemorrhagic stroke were randomized (33/33); 30 BCI and 31 control participants completed the intervention	2 weeks to <1 year after stroke onset	Departments of Rehabilitation Medicine, Huashan Hospital and Jing’an District Central Hospital of Shanghai
S13	Valdés et al., 2017 [26]	Canada	Randomized crossover trial	15 community-dwelling adults with hemiplegia after nontraumatic hemorrhagic or ischemic stroke	>3 months after stroke (eligibility criterion)	University research laboratory
S14	Brunner et al., 2024 [27]	Denmark	Randomized controlled pilot trial	40 patients with severe upper-limb paresis were randomized; 35 were analyzed at the 3-month follow-up (BCI *n* = 15; control *n* = 20)	Subacute stroke; within 60 days after stroke onset	Specialized neurorehabilitation hospital
S15	Guo et al., 2022 [28]	China	Randomized controlled trial	30 chronic stroke patients were randomized equally to BCI-robotic, robotic, or conventional rehabilitation groups (*n* = 10 each)	Chronic stroke; >6 months after a single unilateral stroke	Clinical rehabilitation setting, University of Hong Kong-Shenzhen Hospital
S16	Ambrosini et al., 2019 [29]	Italy/Germany	Prospective first clinical evaluation within an ongoing multicenter randomized trial; uncontrolled analysis	Seven post-acute stroke patients recruited from two clinical sites completed the reported RETRAINER-ARM evaluation	Post-acute stroke; first stroke 2 weeks to 9 months before enrollment	Inpatient and outpatient clinical rehabilitation at two centers
S17	Feingold-Polak et al., 2024 [30]	Israel	Pilot randomized controlled trial	33 post-stroke participants were randomized to socially assistive robot, computer, or usual-care control groups; 26 completed the study	Post-stroke outpatient population with mild to moderate-severe upper-limb impairment	Outpatient unit of the Adi-Negev Rehabilitation Center

**Table 4:** Sensor, system, control, and task characteristics of included studies.

Study ID	Sensor Modality	System/Device	Recognition or Control Approach	Target Task	Rehabilitation Application Level	Multimodal Integration Level
S1	Surface EMG (paretic FDS, FDP, and EDC)	AI-integrated EMG-driven exoskeletal robot hand (MELTIN MMI) with wrist splint and balanced forearm support	EMG patterns for relaxation, finger extension, and finger flexion were calibrated before training; AI identified finger-movement intention from the 3-muscle EMG activity pattern and the robot then assisted movement	Finger flexion/extension; grip-and-release; pinch-and-release; peg pick-and-place and cue-ball transfer tasks	Active-assisted robotic upper-extremity rehabilitation; comparator received passive finger movement with the same robot	sEMG-driven intention detection + robotic mechanical assistance; arm support and patient display were used during task practice
S2	fNIRS and surface EMG	Brite MKIII fNIRS system; BTS FREEEMG 300; bionic soft hand rehabilitation robot glove (Hunan Sirrem Medical Technology Co., Ltd.)	No sensor-based control was reported. fNIRS and sEMG were synchronized measurement modalities; the researcher manually synchronized recording with robot-assisted tasks	Passive grasp/finger movement; mirror-image grasping/finger movement; resistance finger extension and grasping	Robot-assisted hand-function therapy using passive, mirror, and resistance modes; functional role: intention-related physiological assessment; no direct intention decoding or sensor-triggered device control.	Simultaneous fNIRS+sEMG measurement during robot-assisted hand tasks; sensors were used for physiological assessment, not robot control
S3	Surface EMG from the non-paretic upper limb (ECU for wrist extension; EDC for finger extension)	Double-channel electromyographic bridge (EMGB) connected to NMES	MAV/NSS dual coding (MNDC): EMG mean absolute value modulated stimulation pulse duration and number of slope sign changes modulated pulse frequency; contralateral EMG controlled paretic-limb NMES in real time	Wrist extension and hand opening/finger extension during simultaneous bilateral movement	Contralaterally controlled NMES for wrist/finger rehabilitation, added to standard physical and occupational therapy	Contralateral sEMG + NMES + bilateral movement; rhythmic auditory cue and visual observation of the paretic hand were incorporated
S4	EEG for BCI control; fNIRS for mechanism assessment	20-channel Neuracle EEG system; BCI-controlled soft robotic glove (Nanjing Reseader Medical Technology Co., Ltd.); NirSmart fNIRS system	Online motor-imagery classification used mu-suppression/ERD information from C3/C4 with a calibration-derived threshold; successful detection triggered soft-robotic hand assistance	Motor imagery of grasping a cup with the affected hand; repeated grasping task	BCI-controlled soft robotic glove training in addition to conventional rehabilitation; comparator used the same glove without BCI control	EEG-driven robotic/mechanical feedback with visual task/animation feedback; fNIRS was used as an adjunct pre/post cortical assessment
S5	Surface EMG from the non-paretic hand/forearm; Leap Motion hand-motion sensing	STIWELL med 4 FES; Rehabilitation Gaming System (RGS, Eodyne Systems) with Leap Motion sensor	FES of the paretic wrist/finger extensors was triggered when contralateral non-paretic EMG exceeded an individually set threshold; hand motion was captured by Leap Motion and mapped to the virtual avatar	RGS “Bubbles” game: hand opening/closing with reaching and bimanual coordination; FES supported wrist/finger extension	Feasibility and immediate orthotic-support study of serious gaming + contralateral EMG-triggered FES versus serious gaming alone	sEMG + FES + optical hand-motion sensing + VR/serious-game feedback; visual/auditory game feedback was present
S6	11-channel EEG for BCI; sEMG of extensor carpi radialis as a secondary outcome measure	g.USBamp EEG system; VR glasses/virtual hands; brush used for bilateral hand sensory input	Real-time EEG was collected and analyzed; when the EEG pattern corresponded to the preset pattern, the virtual hand displayed the imagined grasping or extending movement. A specific classifier algorithm was not reported	Motor imagery of grasping or extending the right/left hand; classic MI also included upper-limb joint and daily task imagery	Motor-imagery BCI training with sensory and visual feedback versus classic motor-imagery training	EEG + tactile sensory input (brush) + VR visual feedback; sEMG was an outcome measure rather than a control signal
S7	Surface EMG from selected affected-arm muscle pairs; 8-muscle EMG used to identify abnormal co-activation	Myoelectric computer interface (MyoCI) with customized C++ acquisition software and Python computer game	EMG amplitudes from a selected muscle pair were averaged over the preceding 50 ms and mapped to orthogonal cursor components; training reinforced independent activation and reduced abnormal co-activation	Independent activation/decoupling of three abnormal co-activating muscle pairs; isometric or movement-based game control	Game-based myoelectric biofeedback/conditioning for chronic stroke arm rehabilitation	sEMG + real-time visual computer-game cursor feedback; no robotic or electrical-stimulation actuator was used
S8	EEG sensorimotor rhythms for BMI control; EMG and fMRI used for outcome/follow-up assessment	BMI-controlled robotic orthoses for the paretic hand/upper limb	Voluntary desynchronization of ipsilesional EEG sensorimotor rhythms contingently triggered robotic orthosis movement; sham orthosis movement was random and unrelated to the EEG signal	Open/close the fingers; move the paretic upper limb forward/backward (all participants practiced finger opening/closing)	Contingent BMI-robotic orthosis training followed by behavioral physiotherapy; this paper reports 6-month follow-up	EEG + contingent robotic movement providing visual/proprioceptive feedback; EMG and fMRI were assessment modalities, not BMI-control inputs
S9	Surface EMG from paretic biceps and triceps; optical motion capture used for kinematic assessment	Myomo e100 lightweight myoelectric brace; tablet software for therapist adjustment; Raptor-12 motion-capture system for assessment	The user’s movement intention was detected from biceps/triceps EMG; the brace provided adjustable mechanical assistance to paretic elbow movement	Repetitive task-specific upper-extremity practice, including functional tasks and reaching; the device specifically assisted elbow movement	Repetitive task-specific practice integrated with a myoelectric brace versus repetitive task-specific practice alone	sEMG-triggered mechanical assistance integrated with functional task practice; motion capture was used only for kinematic assessment
S10	EEG motor-imagery signals	BCI-controlled hand exoskeleton with computer visual cues	The EEG classifier repeatedly evaluated motor-imagery state online; successful recognition of the imagined hand produced exoskeleton finger extension. In the control condition, exoskeleton movements were independent of motor-imagery EEG	Motor imagery of opening the affected hand; paretic finger extension	Active BCI-controlled hand-exoskeleton training versus passive/sham exoskeleton movement	EEG + online visual feedback + exoskeleton-generated kinesthetic/proprioceptive feedback
S11	Wearable wireless surface EMG; 12-muscle Trigno EMG used for laboratory screening of abnormal co-activation	Wearable Myoelectric Interface for Neurorehabilitation (MINT) with custom wireless EMG acquisition (Myomo, Inc.), laptop, Blender/Python game	Abnormally co-activating muscles were identified by pairwise EMG correlations. During training, EMG envelopes were mapped to orthogonal cursor axes: 2 muscles in 2D, 3 muscles in 3D, or 1 muscle in the 1D sham condition	Independent activation/decoupling of selected abnormal shoulder/elbow muscle sets to move a cursor to visual targets; 2D Reach added reaching cues	Personalized high-dose home-based myoelectric conditioning, with one laboratory session per week; sham used one-muscle cursor control	Wireless sEMG + gamified visual/audiovisual cursor feedback; home and laboratory components were combined
S12	10-channel EEG	Portable BCI system with iRecorder W16/CommercialAmp EEG acquisition and wearable hand exoskeleton for motor feedback	Common spatial pattern (CSP) features with log-variance vectors were classified online by linear discriminant analysis (LDA) to discriminate motor attempt from rest; successful grasp/open intention detection activated robotic assistance	Hand grasping and opening	Functional-oriented portable BCI hand training with robotic feedback versus task-oriented guidance training	EEG intention decoding + wearable hand-exoskeleton motor/kinesthetic feedback; task instructions were provided during training
S13	Kinect 2 motion tracking and JACO2 robotic interaction/force sensing	Two JACO2 6-DOF robotic arms, Kinect 2 camera, personal computer, custom LabVIEW program	Trunk compensation was quantified from motion-tracking data; visual and force feedback used the same compensation-based algorithm, delivered in separate randomized crossover conditions	Symmetric bimanual reaching to virtual targets while reducing compensatory trunk movement	Single-session augmented-feedback training during reaching Functional role: movement/performance feedback; no direct movement-intention decoding.	Visual feedback and force feedback were compared as separate modalities, not delivered simultaneously; kinematic and robotic data were integrated to generate feedback
S14	16-channel EEG for BCI; baseline TMS/EMG for corticospinal-tract assessment	RecoveriX (g.tec): g.Nautilus PRO EEG, patient screen, therapist computer, and bilateral FES devices	First run calibrated the EEG movement detector. In subsequent runs, FES-induced hand movement was provided when left/right motor imagery was discriminated with classification confidence >50%	Motor imagery of left/right hand opening (finger extension and wrist dorsiflexion)	BCI training integrated into usual upper-limb rehabilitation versus standard physiotherapy/occupational therapy	EEG + visual avatar feedback + FES-generated motor/somatosensory feedback + auditory cues; TMS/EMG were assessment modalities
S15	EEG (SSVEP); camera-based real-time hand display; glove pressure sensors	SSVEP-BCI-controlled soft robotic glove	Canonical correlation analysis classified two SSVEP frequencies using 3-s windows updated every 0.25 s; four consecutive identical detections generated the robotic control command	Finger/hand flexion and extension during visual attention to corresponding action stimuli	Direct intention decoding and sensor-triggered robotic-glove control	EEG-based command decoding + camera action-observation feedback + pressure-sensor-supported robotic glove
S16	Surface EMG; exoskeleton angle sensors; RFID-based object/target detection	RETRAINER-ARM passive exoskeleton with EMG-triggered FES and sensorized interactive objects	Voluntary EMG above an individualized threshold triggered FES; joint-angle and RFID data monitored exercise execution and task completion	Reaching, object transfer, lateral elevation, and hand-to-mouth tasks	Sensor-triggered FES with sensor-derived movement/performance monitoring and feedback	sEMG + FES + exoskeleton angle sensing + RFID task monitoring; signals served complementary control and feedback roles
S17	Sensorized everyday objects and kinematic motion measurement within a socially assistive robotic platform	Pepper socially assistive robot or computer-guided gamified upper-limb training platform	Sensor-derived task success and performance information supported instructions, progression, and feedback; no direct movement-intention classifier was reported	Reach-to-grasp, object manipulation, and lifting tasks	Movement/performance feedback and socially assistive task guidance; no direct intention decoding	Object/task sensing + kinematic assessment + audiovisual/social feedback; multimodal information supported training rather than fused intention recognition

**Table 5:** Signal-processing methods, validation, and real-time application of the included studies.

Study ID	Signal-Processing or Recognition Method	Validation Approach	Reported Performance	Real-Time Application
S1	Calibrated three-muscle sEMG pattern classification using an AI-integrated system	Randomized comparison of active-assisted and passive robotic training	Recognition accuracy and response latency not reported	EMG-driven robotic assistance
S2	Synchronized fNIRS and sEMG measurement without a device-control algorithm	Randomized comparison of three robotic training modes	Intention recognition not applicable	No direct recognition or device control
S3	MAV/NSS-based EMG coding for modulation of NMES pulse duration and frequency	Randomized comparison of EMG-bridge and cyclic NMES training	Recognition accuracy and response latency not reported	Contralateral EMG-controlled NMES
S4	Mu-suppression/ERD detection using an individually calibrated threshold	Randomized pilot trial of a BCI-controlled glove	Detection threshold reported; accuracy and latency not reported	Detected motor imagery triggered glove assistance
S5	Individual contralateral EMG threshold combined with optical motion tracking	Randomized crossover feasibility study	Individual threshold reported; response latency not reported	EMG-triggered FES and game feedback
S6	Preset EEG-pattern matching; classifier details not specified	Randomized preliminary BCI study	Classification accuracy and response latency not reported	Detected EEG pattern generated virtual-hand feedback
S7	EMG signals averaged over 50 ms and mapped to two-dimensional cursor movement	Randomized three-group trial	Processing window and cursor mapping reported; latency not reported	Continuous EMG-based game feedback
S8	Online detection of desynchronization in ipsilesional sensorimotor rhythms	Randomized controlled BMI study with follow-up	Classification accuracy and response latency not reported	EEG activity triggered robotic orthosis movement
S9	Intention detection using EMG signals from the paretic biceps and triceps	Randomized subgroup comparison	Recognition accuracy and response latency not reported	EMG-controlled elbow assistance
S10	Online classification of motor-imagery EEG	Blinded randomized multicenter trial	Classification accuracy and response latency not reported	Classified motor imagery triggered exoskeleton movement
S11	Pairwise EMG correlation and EMG-envelope mapping for cursor control	Randomized sham-controlled trial	Cursor-mapping dimensions reported; response latency not reported	Continuous EMG-based game feedback
S12	Common spatial pattern features and linear discriminant analysis	Randomized trial with blinded outcome assessment	Classifier reported; response latency not reported	Detected motor attempt triggered exoskeleton movement
S13	Compensation-detection algorithm using Kinect and robot-interaction data	Randomized crossover comparison of visual and force feedback	Intention-classification performance not applicable	Real-time visual or force feedback without intention decoding
S14	Calibrated motor-imagery classifier using a confidence threshold above 50%	Randomized controlled pilot trial	Classification threshold reported; response latency not reported	Classified motor imagery triggered FES
S15	Canonical correlation analysis of two SSVEP frequencies; 3-s window with 0.25-s update and four-consecutive-detection rule	Three-group randomized controlled trial with post-training and 3-month follow-up	Participant-level BCI accuracy reported and correlated with FMA improvement; command latency not directly reported	SSVEP classification generated real-time robotic-glove commands
S16	Individual EMG threshold for FES triggering; angle-sensor and RFID logic for task monitoring	First seven participants in an ongoing multicenter randomized trial; pre-post feasibility analysis without a concurrent control group	98.2% ± 2.2% of FES tasks were EMG-triggered; active involvement and kinematic measures were reported	Real-time EMG-triggered FES with automated task monitoring
S17	Sensor-based task-success detection and kinematic performance analysis; no intention classifier	Three-group pilot randomized controlled trial	Clinical and kinematic outcomes reported; intention-recognition accuracy and response latency not applicable	Real-time robot/computer instructions and performance feedback without intention decoding

## Data Availability

The original contributions presented in this study are included in the article and Supplementary Materials. Further inquiries can be directed to the corresponding author. The review protocol is registered on the Open Science Framework (https://osf.io/4aku9, accessed on 28 July 2026).

## Supplementary Materials

The following supporting information can be downloaded at: https://www.mdpi.com/article/10.3390/s26216680/s1, Table S1: Database-specific Boolean search strategies; File S1: PRISMA 2020 checklist.

## Abbreviations

The following abbreviations are used in this manuscript:

ARAT	Action Research Arm Test
BCI	Brain–computer interface
BMI	Brain–machine interface
CSP	Common spatial pattern
ECU	Extensor carpi ulnaris
EDC	Extensor digitorum communis
EEG	Electroencephalography
EMG	Electromyography
EMGB	Electromyographic bridge
ERD	Event-related desynchronization
FDP	Flexor digitorum profundus
FDS	Flexor digitorum superficialis
FES	Functional electrical stimulation
FMA	Fugl-Meyer Assessment
fMRI	Functional magnetic resonance imaging
fNIRS	Functional near-infrared spectroscopy
IMU	Inertial measurement unit
LDA	Linear discriminant analysis
MI	Motor imagery
MINT	Myoelectric interface for neurorehabilitation
MNDC	MAV/NSS dual coding
MRC	Medical Research Council
MyoCI	Myoelectric computer interface
NMES	Neuromuscular electrical stimulation
OSF	Open Science Framework
PRISMA-ScR	Preferred Reporting Items for Systematic Reviews and Meta-Analyses extension for Scoping Reviews
RGS	Rehabilitation Gaming System
RTP	Repetitive task-specific practice
SCI	Spinal cord injury
sEMG	Surface electromyography
SRG	Soft robotic glove
TBI	Traumatic brain injury
TMS	Transcranial magnetic stimulation
VR	Virtual reality
CCA	Canonical correlation analysis
RFID	Radio-frequency identification
SAR	Socially assistive robot
SSVEP	Steady-state visually evoked potential
