# Supplementary material for: Sensor-Based Hand and Upper-Limb Movement Intention Recognition in Post-Stroke Rehabilitation: A Scoping Review
Source: Sensors (Basel). 2026 Oct 22;26(21):6680. doi: 10.3390/s26216680 (PMC13765752; doi:10.3390/s26216680)
Supplement: Supplementary file 1 [file sensors-26-06680-s001.zip › Supplementary_Table_S1.pdf]

Supplementary Materials

Sensor-Based Hand and Upper-Limb Movement Intention Recognition in Post-Stroke Rehabilitation: A Scoping Review

Jiwon Shin, Wonjun Oh and Youngjoo Cha

Table S1. Database-specific Boolean search strategies.

Database	Search strategy
PubMed	("Stroke"[Mesh] OR stroke*[tiab] OR poststroke[tiab] OR "post-stroke"[tiab] OR "cerebrovascular accident*"[tiab] OR CVA[tiab] OR hemiplegia[tiab] OR hemiparesis[tiab]) AND ("Upper Extremity"[Mesh] OR "upper limb*"[tiab] OR "upper extremit*"[tiab] OR arm[tiab] OR arms[tiab] OR hand[tiab] OR hands[tiab] OR wrist[tiab] OR finger*[tiab] OR grasp*[tiab] OR reach*[tiab]) AND ("Electromyography"[Mesh] OR "Robotics"[Mesh] OR "Orthotic Devices"[Mesh] OR "Wearable Electronic Devices"[Mesh] OR EMG[tiab] OR sEMG[tiab] OR electromyograph*[tiab] OR myoelectric[tiab] OR "myoelectric control"[tiab] OR "EMG-driven"[tiab] OR "EMG based"[tiab] OR "EMG-based"[tiab] OR EEG[tiab] OR electroencephalograph*[tiab] OR BCI[tiab] OR "brain-computer interface*"[tiab] OR "brain computer interface*"[tiab] OR IMU[tiab] OR "inertial measurement unit*"[tiab] OR acceleromet*[tiab] OR gyroscop*[tiab] OR sensor*[tiab] OR "wearable sensor*"[tiab] OR "inertial sensor*"[tiab] OR camera*[tiab] OR vision[tiab] OR "depth camera*"[tiab] OR "RGB-D"[tiab] OR Kinect[tiab] OR "Leap Motion"[tiab] OR multimodal[tiab] OR "multi-modal"[tiab] OR multisensor[tiab] OR "multi-sensor"[tiab] OR "sensor fusion"[tiab] OR "data fusion"[tiab] OR robot*[tiab] OR exoskeleton*[tiab] OR orthos*[tiab] OR "hand orthos*"[tiab] OR "assistive device*"[tiab] OR "assistive technolog*"[tiab] OR "functional electrical stimulation"[tiab] OR FES[tiab] OR NMES[tiab] OR "intention recognition"[tiab] OR "intent recognition"[tiab] OR "motion intention"[tiab] OR "motor intention"[tiab] OR "movement intention"[tiab] OR "movement classification"[tiab] OR "gesture recognition"[tiab] OR "grasp recognition"[tiab] OR "grasp intention"[tiab] OR "movement prediction"[tiab] OR "movement decoding"[tiab] OR "motor decoding"[tiab] OR "volitional control"[tiab] OR "voluntary control"[tiab] OR "user intention"[tiab] OR "intent-driven"[tiab] OR "intention-based"[tiab])
Embase	(exp stroke/ OR stroke*:ti,ab,kw OR poststroke:ti,ab,kw OR "post-stroke":ti,ab,kw OR "cerebrovascular accident*":ti,ab,kw OR CVA:ti,ab,kw OR hemiplegia:ti,ab,kw OR hemiparesis:ti,ab,kw) AND (exp upper limb/ OR exp arm/ OR exp hand/ OR exp wrist/ OR exp finger/ OR "upper limb*":ti,ab,kw OR "upper extremit*":ti,ab,kw OR arm:ti,ab,kw OR arms:ti,ab,kw OR hand:ti,ab,kw OR hands:ti,ab,kw OR wrist:ti,ab,kw OR finger*:ti,ab,kw OR grasp*:ti,ab,kw OR reach*:ti,ab,kw) AND (exp electromyography/ OR exp electroencephalography/ OR exp brain computer interface/ OR exp robotics/ OR exp orthosis/ OR exp exoskeleton/ OR exp functional electrical stimulation/ OR EMG:ti,ab,kw OR sEMG:ti,ab,kw OR electromyograph*:ti,ab,kw OR myoelectric:ti,ab,kw OR "myoelectric control":ti,ab,kw OR "EMG-driven":ti,ab,kw OR EEG:ti,ab,kw OR BCI:ti,ab,kw OR "brain-computer interface*":ti,ab,kw OR "brain computer interface*":ti,ab,kw OR IMU:ti,ab,kw OR "inertial measurement unit*":ti,ab,kw OR acceleromet*:ti,ab,kw OR gyroscop*:ti,ab,kw OR sensor*:ti,ab,kw OR "wearable sensor*":ti,ab,kw OR camera*:ti,ab,kw OR vision:ti,ab,kw OR "depth camera*":ti,ab,kw OR "RGB-D":ti,ab,kw OR Kinect:ti,ab,kw OR "Leap Motion":ti,ab,kw OR robot*:ti,ab,kw OR exoskeleton*:ti,ab,kw OR orthos*:ti,ab,kw OR FES:ti,ab,kw OR NMES:ti,ab,kw OR multimodal:ti,ab,kw OR "multi-modal":ti,ab,kw OR multisensor:ti,ab,kw OR "multi-sensor":ti,ab,kw OR "sensor fusion":ti,ab,kw OR "data fusion":ti,ab,kw OR "intention recognition":ti,ab,kw OR "intent recognition":ti,ab,kw OR "motor intention":ti,ab,kw OR "movement intention":ti,ab,kw OR "movement classification":ti,ab,kw OR "gesture recognition":ti,ab,kw OR "grasp recognition":ti,ab,kw OR "movement prediction":ti,ab,kw OR "movement decoding":ti,ab,kw OR "motor decoding":ti,ab,kw OR "volitional control":ti,ab,kw OR "voluntary control":ti,ab,kw OR "user intention":ti,ab,kw OR "intent-driven":ti,ab,kw OR "intention-based":ti,ab,kw)
CINAHL	(MH "Stroke+" OR TI stroke* OR AB stroke* OR TI poststroke OR AB poststroke OR TI "post-stroke" OR AB "post-stroke" OR TI "cerebrovascular accident*" OR AB "cerebrovascular accident*" OR TI CVA OR AB CVA OR TI hemiplegia OR AB hemiplegia OR TI hemiparesis OR AB hemiparesis) AND (MH "Upper Extremity+" OR TI "upper limb*" OR AB "upper limb*" OR TI "upper extremit*" OR AB "upper extremit*" OR TI arm OR AB arm OR TI arms OR AB arms OR TI hand OR AB hand OR TI hands OR AB hands OR TI wrist OR AB wrist OR TI finger* OR AB finger* OR TI grasp* OR AB grasp* OR TI reach* OR AB reach*) AND (MH "Electromyography+" OR MH "Electroencephalography+" OR MH "Robotics+" OR MH "Orthoses+" OR MH "Electric Stimulation Therapy+" OR TI EMG OR AB EMG OR TI sEMG OR AB sEMG OR TI electromyograph* OR AB electromyograph* OR TI myoelectric OR AB myoelectric OR TI EEG OR AB EEG OR TI BCI OR AB BCI OR TI "brain-computer interface*"

Database	Search strategy
	OR AB "brain-computer interface*" OR TI "brain computer interface*" OR AB "brain computer interface*" OR TI IMU OR AB IMU OR TI "inertial measurement unit*" OR AB "inertial measurement unit*" OR TI acceleromet* OR AB acceleromet* OR TI gyroscop* OR AB gyroscop* OR TI sensor* OR AB sensor* OR TI "wearable sensor*" OR AB "wearable sensor*" OR TI camera* OR AB camera* OR TI vision OR AB vision OR TI Kinect OR AB Kinect OR TI "Leap Motion" OR AB "Leap Motion" OR TI robot* OR AB robot* OR TI exoskeleton* OR AB exoskeleton* OR TI orthos* OR AB orthos* OR TI FES OR AB FES OR TI NMES OR AB NMES OR TI multimodal OR AB multimodal OR TI "sensor fusion" OR AB "sensor fusion" OR TI "data fusion" OR AB "data fusion" OR TI "intention recognition" OR AB "intention recognition" OR TI "motor intention" OR AB "motor intention" OR TI "movement intention" OR AB "movement intention" OR TI "movement classification" OR AB "movement classification" OR TI "gesture recognition" OR AB "gesture recognition" OR TI "movement decoding" OR AB "movement decoding" OR TI "motor decoding" OR AB "motor decoding" OR TI "volitional control" OR AB "volitional control" OR TI "voluntary control" OR AB "voluntary control")
MEDLINE (Ovid)	(exp Stroke/ OR stroke*.ti,ab,kf. OR poststroke.ti,ab,kf. OR "post-stroke".ti,ab,kf. OR "cerebrovascular accident*".ti,ab,kf. OR CVA.ti,ab,kf. OR hemiplegia.ti,ab,kf. OR hemiparesis.ti,ab,kf.) AND (exp Upper Extremity/ OR exp Arm/ OR exp Hand/ OR exp Wrist/ OR exp Fingers/ OR "upper limb*".ti,ab,kf. OR "upper extremit*".ti,ab,kf. OR arm.ti,ab,kf. OR arms.ti,ab,kf. OR hand.ti,ab,kf. OR hands.ti,ab,kf. OR wrist.ti,ab,kf. OR finger*.ti,ab,kf. OR grasp*.ti,ab,kf. OR reach*.ti,ab,kf.) AND (exp Electromyography/ OR exp Electroencephalography/ OR exp Brain-Computer Interfaces/ OR exp Robotics/ OR exp Orthotic Devices/ OR exp Wearable Electronic Devices/ OR EMG.ti,ab,kf. OR sEMG.ti,ab,kf. OR electromyograph*.ti,ab,kf. OR myoelectric.ti,ab,kf. OR "myoelectric control".ti,ab,kf. OR "EMG-driven".ti,ab,kf. OR EEG.ti,ab,kf. OR BCI.ti,ab,kf. OR "brain-computer interface*".ti,ab,kf. OR "brain computer interface*".ti,ab,kf. OR IMU.ti,ab,kf. OR "inertial measurement unit*".ti,ab,kf. OR acceleromet*.ti,ab,kf. OR gyroscop*.ti,ab,kf. OR sensor*.ti,ab,kf. OR "wearable sensor*".ti,ab,kf. OR camera*.ti,ab,kf. OR vision.ti,ab,kf. OR "depth camera*".ti,ab,kf. OR "RGB-D".ti,ab,kf. OR Kinect.ti,ab,kf. OR "Leap Motion".ti,ab,kf. OR robot*.ti,ab,kf. OR exoskeleton*.ti,ab,kf. OR orthos*.ti,ab,kf. OR "functional electrical stimulation".ti,ab,kf. OR FES.ti,ab,kf. OR NMES.ti,ab,kf. OR multimodal.ti,ab,kf. OR "multi-modal".ti,ab,kf. OR multisensor.ti,ab,kf. OR "multi-sensor".ti,ab,kf. OR "sensor fusion".ti,ab,kf. OR "data fusion".ti,ab,kf. OR "intention recognition".ti,ab,kf. OR "intent recognition".ti,ab,kf. OR "motion intention".ti,ab,kf. OR "motor intention".ti,ab,kf. OR "movement intention".ti,ab,kf. OR "movement classification".ti,ab,kf. OR "gesture recognition".ti,ab,kf. OR "grasp recognition".ti,ab,kf. OR "movement prediction".ti,ab,kf. OR "movement decoding".ti,ab,kf. OR "motor decoding".ti,ab,kf. OR "volitional control".ti,ab,kf. OR "voluntary control".ti,ab,kf. OR "user intention".ti,ab,kf. OR "intent-driven".ti,ab,kf. OR "intention-based".ti,ab,kf.)
PEDro	Because PEDro does not support a full three-block Boolean strategy equivalent to PubMed, Embase, CINAHL, or MEDLINE, a broader rehabilitation search was conducted and records were manually screened for sensor-based intention recognition, control, or feedback relevance. Searches combined the term stroke in the title/abstract field with the neurology subdiscipline and upper-limb body-region filters: stroke AND neurology AND hand or wrist; stroke AND neurology AND forearm or elbow; stroke AND neurology AND upper arm, shoulder or shoulder girdle. Retrieved records were then screened for relevant terms and concepts, including EMG, sEMG, EEG, BCI, sensor, wearable sensor, IMU, camera, Kinect, Leap Motion, robot, orthosis, exoskeleton, FES, NMES, intention recognition, movement classification, movement decoding, and sensor-based feedback.
IEEE Xplore	(stroke OR poststroke OR "post-stroke" OR "cerebrovascular accident" OR CVA OR hemiplegia OR hemiparesis) AND ("upper limb" OR "upper extremity" OR arm OR arms OR hand OR hands OR wrist OR finger OR fingers OR grasp OR grasping OR reach OR reaching) AND (EMG OR sEMG OR electromyography OR electromyographic OR myoelectric OR "myoelectric control" OR "EMG-driven" OR "EMG based" OR "EMG-based" OR EEG OR electroencephalography OR IMU OR "inertial measurement unit" OR accelerometer OR accelerometers OR gyroscope OR gyroscopes OR sensor OR sensors OR "wearable sensor" OR "wearable sensors" OR "inertial sensor" OR "inertial sensors" OR camera OR cameras OR vision OR "depth camera" OR "depth cameras" OR "RGB-D" OR Kinect OR multimodal OR "multi-modal" OR multisensor OR "multi-sensor" OR "sensor fusion" OR "data fusion" OR robot OR robotic OR robotics OR exoskeleton OR exoskeletons OR orthosis OR orthotic OR "hand orthosis" OR "assistive device" OR "assistive devices" OR "assistive technology" OR "assistive technologies" OR "intention recognition" OR "intent recognition" OR "motion intention" OR "motor intention" OR "movement intention" OR "movement classification" OR "gesture recognition" OR "grasp recognition" OR "grasp intention" OR "movement prediction" OR "movement decoding" OR "motor decoding" OR "volitional control" OR "voluntary control" OR "user intention" OR "intent-driven" OR "intention-based") AND (randomized OR randomised OR randomly OR "randomized controlled trial" OR "randomised controlled trial" OR RCT OR trial OR "controlled trial" OR "clinical trial")

The primary database searches were run from database inception to 21 July 2026. A supplementary IEEE Xplore search was conducted on 24 September 2026. Grey literature was not searched. PEDro does not support a full three-block Boolean strategy, so a broader rehabilitation search was run and records were screened manually.

The supplementary IEEE Xplore search was last run on 24 September 2026 and yielded 33 records. After screening and full-text assessment, three additional studies met the eligibility criteria and were included in the review.
